# Capsule Endoscopy for Portal Hypertensive Enteropathy

**DOI:** 10.1155/2016/8501394

**Published:** 2015-12-27

**Authors:** Seong Ran Jeon, Jin-Oh Kim

**Affiliations:** Institute for Digestive Research, Digestive Disease Center, Soonchunhyang University College of Medicine, 59 Daesagwan-ro, Yongsan-gu, Seoul 140-743, Republic of Korea

## Abstract

Portal hypertensive enteropathy (PHE) is a mucosal abnormality of the small bowel that is observed in patients with portal hypertension (PH) and can lead to gastrointestinal bleeding and anemia. The pathogenesis is still not completely understood. The introduction of new endoscopic methods, including capsule endoscopy (CE) or balloon-assisted enteroscopy, has increased the detection of these abnormalities. CE can also serve as a road map for deciding subsequent interventions and evaluating the treatment effect. The prevalence of PHE is reportedly 40–70% in patients with PH. Endoscopic findings can be roughly divided into vascular and nonvascular lesions such as inflammatory-like lesions. Traditionally, PHE-associated factors include large esophageal varices, portal hypertensive gastropathy or colopathy, Child-Turcotte-Pugh class B or C, a history of variceal treatment, and acute gastrointestinal bleeding. More recently, on using scoring systems, a high computed tomography or transient elastography score was reportedly PHE-related factors. However, the prevalence of PHE and its related associated factors remain controversial. The management of PHE has not yet been standardized. It should be individualized according to each patient's situation, the availability of therapy, and each institutional expertise.

## 1. Introduction

Portal hypertension (PH) is defined as an elevated hepatic venous pressure gradient (HVPG) >5 mmHg [1, 2]. When the HVPG is more than 10 mmHg, clinically meaningful PH occurs [3]. The diverse causes of PH can be classified as pre-, intra-, and posthepatic according to the anatomical location of the obstacle to blood flow. Liver cirrhosis (LC) is one of the most common causes of PH. In LC, factors contributing to PH can be divided into increased vascular resistance to portal blood flow at the hepatic microcirculation and increased splanchnic blood flow [4]. PH leads to various mucosal abnormalities of the gastrointestinal (GI) tract, which are named according to the anatomical site such as esophageal or gastric varices (EVs or GVs), portal hypertensive gastropathy or colopathy (PHG or PHC), or portal hypertensive enteropathy (PHE) [5]. PH may induce variceal bleeding, ascites, and hepatic encephalopathy and lead to death. Variceal bleeding accounts for at least 30% of mortality in patients with LC [6].

It has been widely recognized that the prognosis-related major bleeding of PH generally originates from the EVs or GVs. Therefore, the development of capsule endoscopy (CE) and balloon-assisted enteroscopy (BAE) has enabled easy access to the small bowel, while PHE is occasionally the cause of overt GI bleeding or anemia in patients with LC. The mucosal abnormalities of the small bowel in patients with LC and PH are described with the term PHE [7]. The edematous and hyperemic lesions that are reminiscent of inflammatory lesions comprise another definition of PHE [8]. However, the definitions remain unclear. In this era of CE and BAE, the aim of this paper is to review the existing evidence of PHE in terms of its classifications, mechanisms, epidemiology, clinical manifestations, endoscopic findings, PHE-associated factors, and management.

## 2. Classifications

In 2005, De Palma et al. [9] reported a landmark study using CE to study small bowel lesions of patients with PH and anemia. This study aimed to better define the mucosal abnormalities of PHE by classifying it into two grades. Grades 1 and 2 were defined as mucosal inflammatory-like abnormalities (edema, erythema, granularity, friability, and/or spontaneous bleeding) and vascular lesions (cherry-red spots, telangiectasias, angiodysplasia-like lesions, and varices), respectively [9]. Another landmark study by Abdelaal et al. [10] classified PHE into red spots, angioectasias, small bowel varices, and inflammatory-like lesions using CE. The first three types comprised the vascular lesions of PHE. Unlike previous studies, Kodama et al. [11] examined abnormal endoscopic findings using double-balloon enteroscopy (DBE) in patients with PH and divided PHE into villous abnormalities (edema, atrophy, and reddening of villi) and vascular lesions (angiodysplasia-like lesions, dilated/proliferated vessels, and varices). The subclassifications of these lesions are shown in Table 1.

## 3. Etiopathogenic Mechanisms

**Figure 1:**
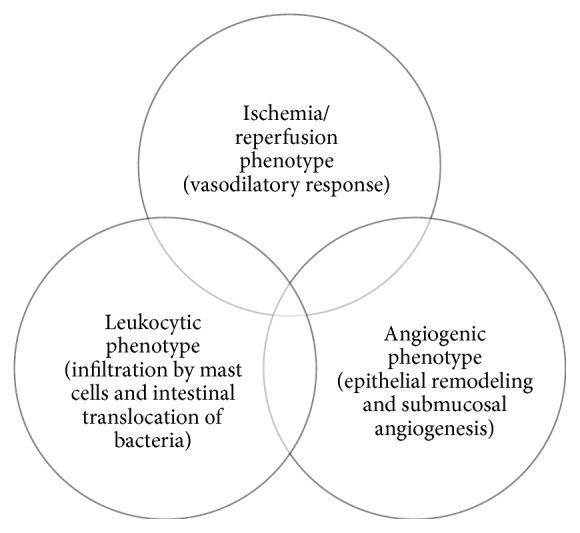
Pathophysiological mechanisms of portal hypertensive enteropathy in a rat model. Modified from Aller et al. [12].

An experimental PH model using portal vein ligated rats reported that splanchnic and/or systemic impairments could have etiopathogenic mechanisms similar to those involved in the posttraumatic inflammatory response [12]. PHE showed three phenotypes during development: ischemia-reperfusion, inflammatory cell infiltration, and angiogenesis. In the ischemia-reperfusion phenotype, PH and venous stasis are associated with mucosal hypoxia, muscularis vasodilation, and arteriovenous shunts opening, which leads to a redistribution of the blood flow within the intestine [12]. Rat mast cell protease-II, a specific marker of rat mucosal mast cell degranulation, plays an important role in the leukocytic phenotype [12] by increasing intestinal permeability, enhancing antigen and bacterial uptake, and inducing bacterial translocation to the mesenteric lymph nodes [13]. Consequently, it promotes mast cell migration and activation in the mesenteric lymph nodes, which induces both mesenteric adenitis and an inflammatory response of the small bowel [14]. Finally, in the angiogenic phenotype, goblet cell hyperplasia, which is considered an alternative characteristic of epithelial remodeling, could be attributed to PHE. This could be responsible for the noted submucosal angiogenesis and muscularis mucosa fibrosis (Figure 1) [12, 15].

## 4. Epidemiology

The reported prevalence of PHE is 18–100%, which shows wide variation (Table 2) [8–10, 16–22]. Most recently, a study by Aoyama et al. reported that the prevalence of PHE was present in 68% of patients with LC [16]. According to data derived from most studies including this study, the prevalence of PHE exceeds 60%. However, when the narrow definition of PHE (edematous and hyperemic lesions that are reminiscent of inflammatory lesions) was used, the prevalence of PHE was reportedly only 18.2% [8], a lower percentage than that reported previously. In our multicenter study that enrolled 45 patients with LC and PH [17], the prevalence of PHE was 40%. This is worthy of notice in the way that deduced the result by using multicenter data based on a large registry including up to 3,000 CE cases.

Although the prevalence of each type of mucosal lesion in PHE also is heterogeneous, vascular lesions including angiodysplasia-like lesions seem to be more common than nonvascular lesions [2, 17]. Small bowel varices and active bleeding were reported in 8.1–38.9% and 5.5–16.6% of cases, respectively [9, 17, 18, 20, 23]. Therefore, active bleeding lesions are not uncommon, and PHE may be considered the cause of meaningful GI bleeding or anemia in patients with LC.

## 5. Clinical Manifestations 

PHE is usually asymptomatic, but chronic GI bleeding and massive bleeding are occasionally reported. Both LC and non-LC causes such as portal vein or splenic vein thrombosis, extrinsic compression (e.g., tumor), venoocclusive disease, and Budd-Chiari syndrome [4] can cause PH; such cases are usually diagnosed by CE or BAE. In patients with PH and/or LC, the most common indication for CE is obscure gastrointestinal bleeding (OGIB) and a high diagnostic yield of 71.1–89.5% has been reported [17, 23]. CE can be used to identify potential bleeding sources and could have diagnostic utility in patients with PH and OGIB.

Follow-up data for patients with PHE are limited. In our multicenter study [17], the rebleeding rate among patients who were followed was not low (46.7%). Among them, small bowel lesions were found in more than 70% of patients, although no death due to small bowel bleeding occurred. This could be inferred from the fact that there is a need for further treatment or intensive care in patients who are diagnosed with PHE using CE.

## 6. Endoscopic Findings 

**Figure 2:**
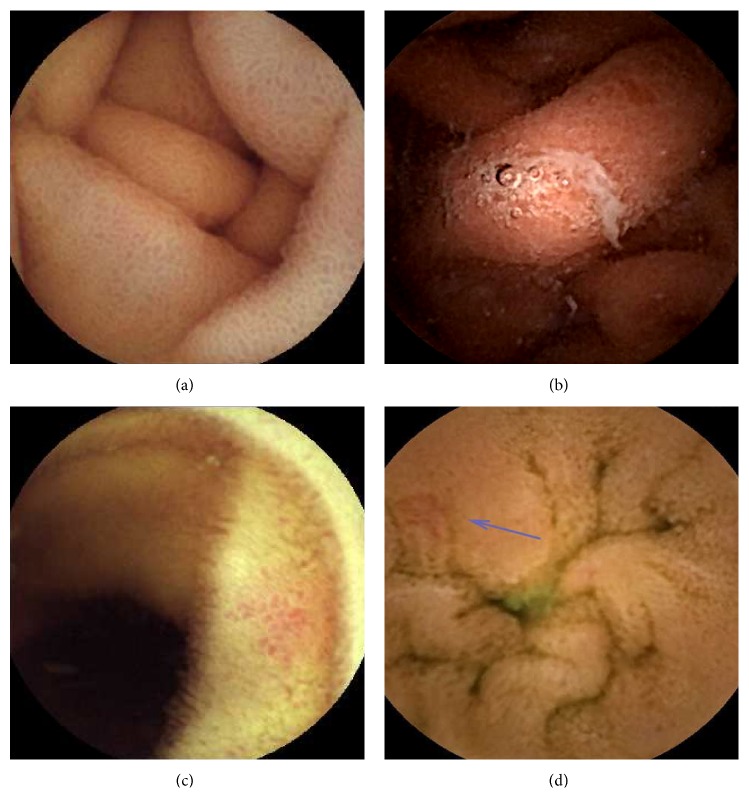
Portal hypertensive enteropathy (inflammatory-like abnormalities, grade 1) found by capsule endoscopy. (a) Reticulate pattern of the mucosa, (b) edematous mucosa with reticulate pattern, (c) hyperemic change with mucosal granularity, and (d) salmon roe appearance with hyperemia (blue arrow).

**Figure 3:**
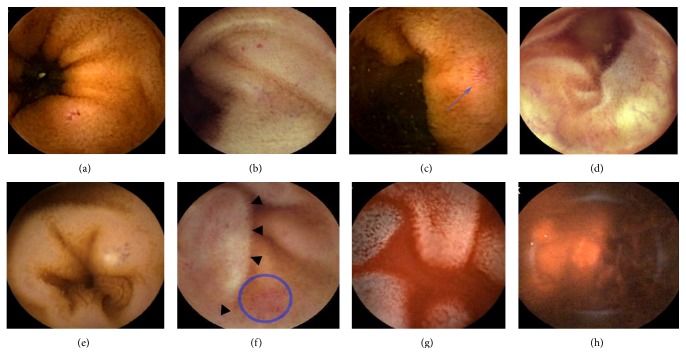
Portal hypertensive enteropathy (vascular lesions, grade 2) found by capsule endoscopy. (a) Red spots, (b) red spots with mucosal granularity, (c) angioectasia (blue arrow), (d) varix with bluish coloration, (e) small saccular varix with a bluish color change and shinning surface, (f) saccular-shaped varix (black arrow head) with shiny and bluish coloration, and hyperemia (blue circle), (g) varix with bluish coloration and fresh blood in the small bowel, and (h) active bleeding due to small bowel varices.

As previously mentioned, PHE can be classified into mucosal inflammatory-like abnormalities (Figure 2) such as edema, erythema, granularity, and friability and vascular lesions (Figure 3) including cherry-red spots, telangiectasias, angiodysplasia-like lesions, and varices [9]. A single-center study reported that areas of mucosa with a reticulate pattern were noted significantly more frequently in patients with PH and/or LC than in those without PH [8]. In another study, a mosaic pattern and severe hyperemia/erythema of the small bowel mucosa were said to have a salmon roe appearance [24].

Red spots that are less well defined or very small are considered insignificant. Angiodysplasia is called angioectasia or vascular ectasia. Such lesions are flat or slightly raised above the mucosal surface, red in color, and have a sharply circumscribed fern-like appearance [25]. Angiodysplasia-like lesions of patients with PHE can be difficult to differentiate from angioectasia of the small bowel secondary to degenerative lesions [26]. The former present as multiple and diffuse, while the latter are usually fewer in number, smaller, and less widely distributed [8]. Such lesions are usually reported in patients with chronic kidney disease, with aortic stenosis, or of advanced age. Small bowel varices were defined as circumferential raised venous lesions that resemble classic images of EVs or GVs [23] and have a typical serpiginous appearance with or without bluish coloration. In fact, it may not be diagnosed due to its diverse appearance. Portal hypertensive polypoid enteropathy characterized by protruding red bumps in the small bowel of a patient with LC is rarely reported [27].

However, the diagnosis can be difficult because some endoscopic findings are nonspecific. The differential diagnosis includes ischemia, inflammatory bowel disease, radiation changes, celiac disease, arteriovenous malformations, and hereditary hemorrhagic telangiectasia.

## 7. Scoring Systems 

Before the study by Kodama et al. [11] using DBE, there was no system for scoring the severity of endoscopic abnormalities in patients with LC and PHE. These authors proposed a scoring system with a maximum of six points. Each category was subclassified into three further subcategories, and every positive finding of these six subcategories was given. However, their scoring system was not significantly associated with any of the clinical variables except for presence of ascites.

More recently, Abdelaal et al. [10] reported a scoring system for PHE using CE. Each of these four lesions (inflammatory-like lesions, red spots, angioectasias, and small bowel varices) was worth two points if it was more than two lesions and one point if it was not. Moreover, using transient elastography (TE), a noninvasive ultrasound-based test, to quantify liver fibrosis, they found that a high TE score was significantly related with the PHE score and concluded that the noninvasive and inexpensive TE method helped clinicians detect PHE presence and severity in patients with LC.

We recently suggested a new scoring system to evaluate whether the abdominal computed tomography (CT) findings were related to PHE diagnosed by CE [17]. The six findings are described in Table 3. Each of these six findings was worth one point, and the points were totaled to give a score from 0 to 6 points. On univariate analysis, Child-Turcotte-Pugh (CTP) score were significantly associated with PHE. However, on multivariate analysis, only a high CT score was significantly related to PHE in patients with LC and PH. This is probably due to the components of the CT score, which are well-known clinical indicators of PH [28]. Our result reflects that a proposed new CT scoring system could better select patients who should undergo CE because of the suspected presence of PHE, especially patients with PH and OGIB. We are planning to begin a prospective multicenter study to overcome limitations of a small number of patients as well as a retrospective design to obtain a more standardized scoring system.

## 8. Risk Factors Associated with PHE 

To date, these risk factors include a large EVs, PHG, PHC, CTP class B or C, a history of endoscopic variceal injection sclerotherapy or ligation, a history of acute GI bleeding, a high TE score, and a high CT score [8–10, 18–20]. The clinical risk factors associated with PHE are summarized in Table 2.

Most recently, Mekaroonkamol et al. [2] sought definitive major predictors of PHE because of the heterogeneity, inconsistency, and complexity of its related risk factors. They found that the PHE-related factors were similar to those previously reported, including CTP class B or C, portosystemic shunts (PSs), ascites, portal thrombosis, EVs, and PHG. On multivariate analysis, however, only PS was an independent risk factor to predict PHE. PSs, which act as bypasses to compensate for PH [29], develop when the portal pressure exceeds 10 mmHg, which acts as an indicator of severe PH and EVs exacerbation [30, 31]. They suggested that PSs reflected the status of liver function.

Interestingly, in a study of PHE before and after EVs obliteration, the authors found that PHE increased significantly from 6.6% before obliteration to 46.7% after variceal obliteration [32]. This is similar to the results of another study [33] in which the authors found higher vascular endothelial growth factor (VEGF) tissue expression in the small bowel biopsies after variceal obliteration. The circulating VEGF level in patients with LC has been shown to reflect increased PH or decreased hepatic regenerative activity [34]. Thus, variceal obliteration may be the worsening factor.

## 9. Management 

Although CE is the preferred initial diagnostic modality for identifying bleeding sources in the small bowel, it has major limitations, including lacking the capability to repeatedly examine the lesions and perform therapeutic intervention [22, 35]. However, CE can serve as a road map to decide subsequent interventions and evaluate the treatment effect. PHE has been attempted as therapeutic intervention in 5–38.9% of patients with PH and chronic active bleeding [9, 17, 20]; however, standardized therapeutic guidelines for symptomatic PHE lesions do not exist.

The benefit of medical treatment such as nonselective beta-blockers or somatostatin has not yet been extensively studied in PHE. Interestingly, single case reports have described that thalidomide (100 mg/day), which was able to suppress tumor necrosis factor-alpha and VEGF tissue effects, effectively normalized hemoglobin levels without the need for transfusion [36]. In CE findings performed after treatment, clear lesion regression was observed. However, controlled trials are needed to confirm this effect and evaluate the therapeutic safety and ideal duration.

Other available therapeutic options include endoscopic treatment using BAE, radiological interventions, and surgery. Argon plasma coagulation and/or hemoclips can be used to achieve hemostasis [10, 17, 20, 37]. However, the treatment of small bowel varices should be approached using various modalities. Radiological interventions such as percutaneous coil embolization [38] or transjugular intrahepatic portosystemic shunt (TIPS) placement [39] and variceal injection sclerotherapy using BAE [40] can effectively treat large varices. A recent study including 15 patients with LC and PH [41] evaluated the effects of TIPS on portal venous pressure decompression for PHE detected by CE in patients with LC and PH. The authors found that portal venous pressure was decreased and PHE was attenuated after TIPS. This meaningful study demonstrated the effects of TIPS by CE before and after treatment. Otherwise, surgical interventions such as portosystemic surgical shunt, small bowel resection, or surgical variceal ligation can be performed [42–44]. Regardless of treatment type, CE can be used to recognize the need for specific medical treatment or further interventions and evaluate the posttherapeutic effect. Treatments for PHE should be individually tailored to each patient's clinical situation, available therapy, and local expertise.

## 10. Conclusions

The introduction of CE has enabled the identification of PHE as a potentially significant complication of PH. According to recent studies, the prevalence of PHE is 40–70%. In patients with LC and OGIB, PHE can provide a possible cause for GI blood loss. However, the prevalence and associated factors of PHE should be interpreted considering limitations such as small numbers of patients, heterogeneous designs, the use of various CE criteria, and different interpretations and treatment effects. Despite the development of several proposed scoring systems as part of the effort to find PHE-related risk factors, none has been standardized or validated. Larger well-designed prospective trials are needed to clarify the definition and classification of PHE and validate the existing scoring systems. Since there are currently no standardized treatment guidelines for symptomatic PHE, various therapeutic modalities can be considered for its treatment. Despite its inherent disadvantages, CE is a useful method for identifying treatable lesions in patients with PHE and can help optimize treatment on a case-by-case basis.

## Figures and Tables

**Table 1:** Classifications of endoscopic abnormalities in patients with portal hypertensive enteropathy.

Year	First author	Classification
2005	De Palma [9]	Inflammatory-like lesions (grade 1) Edema, erythema, granularity, friability, and/or spontaneous bleeding Vascular lesions (grade 2) Cherry-red spots, telangiectasias, angiodysplasia-like lesions, and varices

2008	Kodama [11]	Villous lesions Edema of villi Atrophy of villi Reddening of villi Vascular lesions Angiodysplasia-like lesions Red spots, vascular spiders, and lymphoid follicles with dilated vessels Dilated/proliferated vessels Tree-like dilated vessels and coil-like fine vessels Varices

2010	Abdelaal [10]	Red spots Angioectasias Small bowel varices Inflammatory-like lesions

**Table 2:** Prevalence of and factors related to portal hypertensive enteropathy using capsule endoscopy.

Year	First author	Design	Indication	Comparison (number)	Prevalence (%)	Related factors
2015	Aoyama [16]	Retrospective, single	OGIB	PHE versus non-PHE (91 versus 43)	68 versus 0	Univariate analysis: CTP class B or C, PSs, ascites, portal thrombosis, EVs, PHG Multivariate analysis: PSs
2014	Jeon [17]	Retrospective, multicenter	OGIB	PHE versus non-PHE (18 versus 27)	40 versus 0	Univariate analysis: CTP class C, high CT score Multivariate analysis: high CT score
2010	Abdelaal [10]	Prospective, single	OGIB	LC with PH versus non-LC with PH (31 versus 29)	67.7 versus 6.9	High TE score, CTP class B or C, large EVs, PHG, history of EIS/EVL
2010	Akyuz [18]	Prospective, single	OGIB	LC with PH versus non-LC with PH (14 versus 7)	92.8 versus 85.7	No related factors
2009	Kovács [8]	Retrospective, two-hospital	OGIB	LC with PH versus non-LC (11 versus 22)	18.2 versus 0	—
2008	Goulas [19]	Prospective, single	OGIB	LC with PH versus non-LC (35 versus 70)	65.7 versus 15.7	Severe PHG
2008	Figueiredo [20]	Prospective, single	—	PH versus non-PH (36 versus 30)	69 versus 3	History of acute GI bleeding
2005	De Palma [9]	Prospective, single	OGIB	LC with PH versus IBS (37 versus 34)	67.5 versus 0	≥Gr 2+ varices, PHG, PHC, CTP class C

OGIB: obscure gastrointestinal bleeding; CE: capsule endoscopy; PHE: portal hypertensive enteropathy; CTP: Child-Turcotte-Pugh; CT: computed tomography; DBE: double-balloon enteroscopy; PSs: portosystemic shunts; EVs: esophageal varices; PHG: portal hypertensive gastropathy; LC: liver cirrhosis; PH: portal hypertension; LD: liver disease; TE: transient elastography; EIS/EVL: endoscopic variceal injection sclerotherapy or ligation; GI: gastrointestinal; Gr: grade; PHC: portal hypertensive colopathy.

**Table 3:** Currently available scoring systems using various modalities.

Year	First author	Scoring modality	Method	Each type
2014	Jeon [17]	CT	One point for each type of CT finding (a maximum score: six points)	(1) EVs or GVs (2) Other collateral circulations (e.g., periumbilical varices) (3) PHG or PHC (4) Portal hypertensive cholecystopathy (5) Splenomegaly (6) Ascites

2010	Abdelaal [10]	CE	One point for each type of lesion; two points for each type if it is multiple (>2 lesions) (a maximum score: eight points)	(1) Red spots (2) Angioectasias (3) Small bowel varices (4) Inflammatory-like lesions

2008	Kodama [11]	DBE	One point for each type of lesion (a maximum score: six points)	(1) Edema of villi (2) Atrophy of villi (3) Reddening of villi (4) Angiodysplasia-like lesions (5) Dilated/proliferated vessels (6) Varices

CT: computed tomography; EVs: esophageal varices; GVs: gastric varices; PHG: portal hypertensive gastropathy; PHC: portal hypertensive colopathy; CE: capsule endoscopy; DBE: double-balloon enteroscopy.
